# Characterization of the circRNA–miRNA–mRNA Network to Reveal the Potential Functional ceRNAs Associated With Dynamic Changes in the Meat Quality of the Longissimus Thoracis Muscle in Tibetan Sheep at Different Growth Stages

**DOI:** 10.3389/fvets.2022.803758

**Published:** 2022-04-01

**Authors:** Gaoliang Bao, Fangfang Zhao, Jiqing Wang, Xiu Liu, Jiang Hu, Bingang Shi, Yuliang Wen, Li Zhao, Yuzhu Luo, Shaobin Li

**Affiliations:** Gansu Key Laboratory of Herbivorous Animal Biotechnology, Faculty of Animal Science and Technology, Gansu Agricultural University, Lanzhou, China

**Keywords:** circRNA, Tibetan sheep, ceRNA, muscle development, meat quality

## Abstract

Circular RNAs (circRNAs) have a regulatory role in animal skeletal muscle development. In this study, RNA sequencing was performed to reveal the temporal regularity of circRNA expression and the effect of the circRNA–miRNA–mRNA ceRNA regulatory network on the meat quality of longissimus thoracis (LT) muscle in Tibetan sheep at different growth stages (4 months old, 4 m; 1.5 years old, 1.5 y; 3.5 years old, 3.5 y; 6 years old, 6 y). There were differences in the carcass performance and meat quality of Tibetan sheep at different ages. Especially, the meat tenderness significantly decreased (*p* < 0.05) with the increase of age. GO functional enrichment indicated that the source genes of the DE circRNAs were mainly involved in the protein binding, and myofibril and organelle assembly. Moreover, there was a significant KEGG enrichment in the adenosine 5′-monophosphate (AMP)-activated protein kinase (AMPK) signaling pathway, as well as the calcium signaling pathway, regulating the pluripotency of the stem cells. The circRNA–miRNA–mRNA ceRNA interaction network analysis indicated that circRNAs such as circ_000631, circ_000281, and circ_003400 combined with miR-29-3p and miR-185-5p regulate the expression of *LEP, SCD*, and *FASN* related to the transformation of muscle fiber types in the AMPK signaling pathway. The oxidized muscle fibers were transformed into the glycolytic muscle fibers with the increase of age, the content of intramuscular fat (IMF) was lowered, and the diameter of the muscle fiber was larger in the glycolytic muscle fibers, ultimately increasing the meat tenderness. The study revealed the role of the circRNAs in the transformation of skeletal muscle fiber types in Tibetan sheep and its influence on meat quality. It improves our understanding of the role of circRNAs in Tibetan sheep muscle development.

## Introduction

Tibetan sheep is the dominant livestock resource in the Qinghai–Tibet Plateau, raised at an altitude above 3,000 m on the Qinghai–Tibet Plateau with high altitude, low temperature, thinner oxygen, and strong UV radiation. Tibetan sheep meat is loved by consumers who like mutton for its unique flavor (1, 2). Present studies are mainly focused on improving the meat quality and growth performance of Tibetan sheep. Previous studies have identified the gene *FGFR1* to be involved in the growth-related traits, and henceforth, it was used as the genetic marker for improving the growth traits of the Tibetan sheep (3) The polymorphisms in *SIRT1* and *SIRT2* regulate the growth-related traits in Tibetan sheep (4). The quantitative genetic analysis revealed *IRS1* to be associated with the growth-efficiency traits possibly useful for marker-assisted selection in Chinese indigenous sheep (5). Previous studies have also identified stallfeeding to be appropriate for producing Tibetan mutton (6). Mammalian skeletal muscle is a heterogeneous tissue, composed of various muscle fibers exhibiting different physiological and metabolic properties, such as glycolysis, oxidative metabolism, and contraction. The skeletal muscle is the major component of body mass accounting for approximately 50% of the body mass in mammals (7). The skeletal muscle growth and development directly influence meat quality and meat yield. Meanwhile, the composition of the different muscle fiber types has been mainly related to the IMF, meat tenderness, water holding capacity (WHC), flavor and juiciness, and other indicators (8). Therefore, exploring the mechanism of skeletal muscle growth and development is very important for improving the growth rate and meat quality of Tibetan sheep.

Circular RNAs (circRNAs) are covalently closed endogenous non-coding RNA molecule, and which are involved in many cellular and developmental processes in the eukaryotic cells (9, 10). circRNA molecules have a closed circular structure, which is generated from back-splicing reactions of linear RNA, and have significant characteristics like the absence of a free 3′ end tail and 5′ end cap structure, and they cannot be easily degraded by exonuclease in cells and are highly stable (11). The latest research findings found circRNAs as not only a byproduct of splicing during mRNA maturation but also as an important product of pre-mRNA processing. Moreover, the mechanism of circRNA processing is competitive with mRNA processing (12). The function of circRNAs is specific to various tissue and developmental stages, and previous studies have revealed their importance in skeletal muscle development and intramuscular fat deposition (13–15). The circRNA has a variety of biological regulatory functions, serving as a sponge for specific miRNA to negatively regulate the activity of miRNA through the competitive endogenous RNAs (ceRNA) network, and reduce the miRNA-mediated gene silencing (9). The ceRNAs include pseudogenes, lncRNAs, circRNAs, and mRNAs. These RNAs with the same miRNA response element (MRE) are capable of competing for miRNAs binding, thereby the expression of the target gene transcripts was interacted and regulated (16). Li et al. (17) found that circ_0015885 served as a sponge of miR-23b in the skeletal muscle of pigs and further regulates the expression of SESN3. Peng et al. (18) found circSNX29 sponged miR-744, activated the Wnt5a/Ca^2+^ signaling pathway, promoted the differentiation of bovine myoblasts, and inhibited cell proliferation. Zheng et al. (19) found that silencing circHIPK3 could significantly inhibit the growth of human cells. Furthermore, circRNAs are also important for transcription and post-transcriptional gene expression regulation, alternative splicing, protein coding, and protein decoys (20). However, the transformation mechanism of the LT muscle fiber types by ceRNAs and its influence on meat quality in Tibetan sheep at different growth stages remains unclear.

There were a lot of studies that have described circRNA in humans (17, 21), animals (22, 23), plants (24, 25), and microorganisms (26) using an RNA-Sequencing approach. Yan et al. (27) have identified a total of 5,177 circRNAs in the longissimus dorsi muscle of the Kazakh cattle and Xinjiang cattle, and have found the process of muscle development was related to the differentially expressed circRNAs (DE circRNAs). Li et al. (28) have identified a total of 6,113 circRNAs in the embryonic and adult Kazakh sheep, It has been found that circ_0000385 possesses multiple binding sites with miR-143, miR-133, and miR-23 related to muscle development. Shen et al. (29) found that DE circRNAs such as circRNA41 in skeletal muscle of pigs might be involved in the expression of the oxidized muscle fibers and glycolytic muscle fiber-related genes. There were many studies on the regulation of circRNA on livestock. However, there are no studies on the circRNA research of Tibetan sheep muscle development. A total of 16 Tibetan sheep in four growth stages were selected in this study based on the differences in the slaughter performance and meat quality, and the differences of LT muscle growth and development at different growth stages were analyzed. The function of the circRNA and the mechanism of the circRNA–miRNA–mRNA ceRNA regulatory network in the LT muscle development of Tibetan sheep were further analyzed using an RNA sequencing approach, the mechanism of circRNA in the transformation of the oxidized muscle fibers and glycolytic muscle fibers in Tibetan sheep were explored with the increase of age, to explain the new molecular mechanism of Tibetan sheep muscle growth and development and individual growth.

## Materials and Methods

### Ethics Statement

The animal study was reviewed and approved by the Faculty Animal Policy and Welfare Committee of Gansu Agricultural University (Ethic approval file No. GSAU-Eth-AST-2021-001).

### Animals and Muscle Sampling

Sixteen healthy female Tibetan sheep were randomly selected from the same pasture of Haiyan County, Qinghai Province, China (3,500 m above sea level), including 4 m (*n* = 4), 1.5 y (*n* = 4), 3.5 y (*n* = 4), and 6 y (*n* = 4). Also, 4 m, 1.5 y, 3.5 y, and 6 y represent the lambs, the pubertal sheep, the adult sheep, and the old sheep, respectively. All sheep were provided with the same nutrition and were raised under similar environmental conditions with natural light and free access to food and water. Then, four Tibetan sheep from each growth stage were weighed and immediately slaughtered humanely following the Islamic practice (exsanguinated, peeled, and split down the midline according to standard operating procedures). The experiment was conducted in accordance with the guidelines of the National Institutes of Health guide for the care and use of laboratory animals (NIH Publications No. 8023, revised 1978), and the animal welfare and conditions were considered for using the experimental animals. The initial weight and hot carcass weight were recorded, and used to calculate dressing percentage (= hot carcass weight / live weight × 100%). The carcasses were placed in a chilling room at 4°C before sampling for meat quality traits. LT muscle (from 12th thoracic vertebrae to 5th lumbar vertebrae) samples from the left half carcass were collected from the four different growth stages after slaughtering and immediately frozen in liquid nitrogen and stored at −80°C until RNA isolation was performed.

### Meat Quality Measurements

The shear force was measured according to the method of Honikel (30). Forty-eight hours after slaughter, the samples were cooked with a plastic bag in a water bath at 75°C to reach an internal temperature of 70°C and then cooled down to room temperature. From each sample, five slices with a diameter of 1.27 cm were cut from each sample with a cylindrical core drill, and the slices were cut perpendicular to the fiber direction by a shearing device (C-LM3B; Runhu Instrument Co., Ltd., Guangzhou, China). The intramuscular fat content (IMF) was measured using the Soxhlet extraction method with solvent (petroleum ether) and expressed as a weight percentage of wet muscle tissue (31), with three replicates for each sample (32). The crude protein content was measured according to the AOAC (31) procedure, with three replicates for each sample.

### RNA Extraction, Library Construction, and Sequencing

Total RNAs were extracted from the LT muscle samples of Tibetan sheep using Trizol reagent kit (Invitrogen, Carlsbad, CA, USA) according to the manufacturer's protocol. The quality and integrity (RIN values) of RNA were assessed using an Agilent 2100 Bioanalyzer (Agilent Technologies, Palo Alto, CA, USA) and checked using RNase free agarose gel electrophoresis. After extraction, the total RNAs were treated with RNase R to degrade the linear RNAs, and purified using the RNeasy MinElute Cleanup Kit (Qiagen, Venlo, The Netherlands). Next, a strand-specific library was constructed using the VAHTS Total RNA-seq (H/M/R) Library Prep Kit (Vazyme, Nanjing, China) for Illumina following the manufacturer's instructions. Briefly, rRNAs were removed to retain circRNAs. The enriched circRNAs were fragmented into short fragments by using fragmentation buffer and reverse transcribed into cDNA with random primers. Second-strand cDNA were synthesized by DNA polymerase I, RNase H, dNTP (dUTP instead of dTTP), and buffer. Next, the cDNA fragments were purified with VAHTSTM DNA Clean Beads (Vazyme, Nanjing, China), end repaired, poly(A) added, and ligated to Illumina sequencing adapters. Then second-strand cDNA was digested using the UNG (Uracil-N-Glycosylase). The digested products (200–300 bp) were purified with VAHTSTM DNA Clean Beads; the digested products were size-selected and PCR amplified to generate RNA-seq libraries, and sequenced using Illumina Novaseq60000 by Gene Denovo Biotechnology Co. (Guangzhou, China).

### Identification of circRNA

The low-quality reads, including reads with adapters, reads bearing >10% of unknown bases (N), and reads with >50% of low-quality (Q-value ≤ 20) bases, were discarded to acquire clean reads. The resulting high-quality reads were mapped to the sheep Oar_v1.0 reference genome using TopHat2 (version 2.1.1), respectively. After aligning with the reference genome, the reads that could be mapped to the genomes were discarded, and the unmapped reads were then collected for circRNA identification. Then, the 20 mers from both ends of the unmapped reads were extracted and aligned to the reference genome to find unique anchor positions within splice site. Anchor reads that aligned in the reversed orientation (head to tail) indicated circRNA splicing and then were subjected to find_circ (6) (version 1) to identify circRNAs. The anchor alignments were then extended such that the complete read aligns and the breakpoints were flanked by GU/AG splice sites. A candidate circRNA was called if it was supported by at least two unique back spliced reads at least in one sample.

### Functional Annotation and Enrichment Analysis of circRNA Source Genes

The previous studies demonstrated that one of the functions of CircRNAs was realized through regulating the expression of its source gene (33). The source gene is the original gene of a circRNA. Functional enrichment analysis of source genes was performed to identify the main functions of these source genes of circRNAs. To analyze the potential biological functions of circRNAs, the Gene Ontology (GO) annotation as well as Kyoto Encyclopedia of Genes and Genomes (KEGG) pathway analysis of circRNA source genes was performed using the Gene Ontology database (http://www.geneontology.org/), and KEGG was the major public pathway-related database (http://www.kegg.jp/kegg/).

### Quantification of circRNA Abundance and Differentially Expressed circRNAs

The expression abundance of the identified circRNAs was computed by reads per million mapping (RPM) method. To identify differentially expressed circRNAs between every two age groups, the edgeR package (version 3.12.1) (http://www.r-project.org/) was used. We identified circRNAs with a fold change ≥2 and *p* < 0.05 in a comparison between the samples or groups as significant differentially expressed circRNAs.

### Construction of the ceRNA Regulatory Network

Based on our previous Illumina HiSeq miRNA and mRNA sequencing data from the same samples, after extraction of total RNA with Trizol kit (Invitrogen, Carlsbad, CA, USA), polyacrylamide gel electrophoresis (PAGE) was used to enrich RNA molecules of 18–30 bp in size and sequencing for miRNA. After total RNA was extracted, rRNAs were removed to retain mRNAs. The enriched mRNAs were fragmented into short fragments by using fragmentation buffer and reverse transcribed into cDNA with random primers. The digested products were size selected by agarose gel electrophoresis, PCR amplified for mRNA sequencing. The circRNA–miRNA–mRNA ceRNA regulatory network was constructed as following ceRNA theory (11): (1) Expression correlation between mRNA–miRNA or circRNA–miRNA was evaluated using the Spearman rank correlation coefficient (SCC). Pairs with SCC <-0.7 were selected as co-expressed negatively circRNA–miRNA pairs or mRNA–miRNA pairs. (2) Expression correlation between circRNA and mRNA was evaluated using the Pearson correlation coefficient (PCC). Pairs with PCC >0.9 were selected as co-expressed circRNA–mRNA pairs. (3) The hypergeometric cumulative distribution function test was used to analyze whether the common miRNA sponges between the two genes were significant. As a result, only the gene pairs with a *p* < 0.05 were selected. The ceRNA network related to muscle development was visualized by Cytoscape 3.7.1.

### RT-qPCR Analysis and circRNA Sequencing

The total RNA was extracted from the LT muscle samples of Tibetan sheep with TRIzol reagent (Invitrogen, Carlsbad, CA, USA), which was used for RNA-Seq and synthesize cDNA using a RT-PCR Kit (Takara, Dalian, China). Twelve divergent primers used for amplification of circRNA-specific back splice junctions were designed (Table 1). PCR products were confirmed by 1.5% agarose gel electrophoresis and bands were extracted and subjected to Sanger sequencing. The same 12 circRNAs were selected for verifying the reliability of the RNA-Seq by calculating the relative expression levels using the real-time quantitative PCR (RT-qPCR). The RT-qPCR were conducted in triplicates using 2 × ChamQ SYBR qPCR Master (Vazyme, Nanjing, China) on an Applied Biosystems QuantStudio 6 Flex (Thermo Lifetech, MA, USA). The relative expression levels of these genes were analyzed using the 2^−ΔΔCt^ method. Sheep β-actin was used as an internal reference gene.

**Table 1:** A list of primers used in the RT-qPCR.

**CircRNAs**	**Forward (5^′^ → 3^′^)**	**Reverse (5^′^ → 3^′^)**
circ_000154	GAGGCTCACAATCGTTCA	TCCAGGGCTTAGACTTCG
circ_000747	AACCCAGCATCAAATCCA	CCAACAAAGATCGGCTCA
circ_001179	TGCACTTCATCCGCTTCC	GGTTCTCCACCTCCTCCTG
circ_001848	ACTCAGGACCTAAGCGATGT	GGGAATCAGTTCCAGCAT
circ_003667	TCCCAGTGCTGCCCTTAT	TTCCTCCATGTTCAGTTGC
circ_008215	CCCTCCCGTCAGTTTCAC	GGGCTTAGCTTTCGCACA
circ_008292	GCCTGGCTCTACAACTCC	TGGCCTCTGTCTCGATTT
circ_008537	CATTGCTCGCTTCAGTCC	TTCAGCCAGGTGCTTCTT
circ_009087	TCCAGCAAGTTATCCAGT	CAAAGGGTCCAAATGTTA
circ_010337	ATTGCTGGAATCGTTGGA	AAAGCCTCTTGGAGTAGTT
circ_010435	CACCACCCGCACCAAGAC	GGCCACATCCAGGCAAAT
circ_011286	TATGGTTATCACTGCGTTCC	GCTTCACCGTCATCGTCA
β-actin	AGCCTTCCTTCCTGGGCATGGA	GGACAGCACCGTGTTGGCGTAGA

### Correlation Analysis

To further verify the function of the circRNAs in the LT muscle growth and development in Tibetan sheep at different ages, the Pearson correlation analysis was performed between 12 circRNAs and slaughter performance and meat quality. The two-tailed test was used for correlation analysis.

### Statistical Analysis

All statistical analyses were performed using IBM SPSS 22.0 (SPSS, Inc., Chicago, IL, USA). The differences between the mean values were compared by Duncan's multiple range test (*p* < 0.05). Each experiment was replicated at least thrice.

## Results

### Slaughter Performance and Meat Quality of Tibetan Sheep

**Figure 1:**
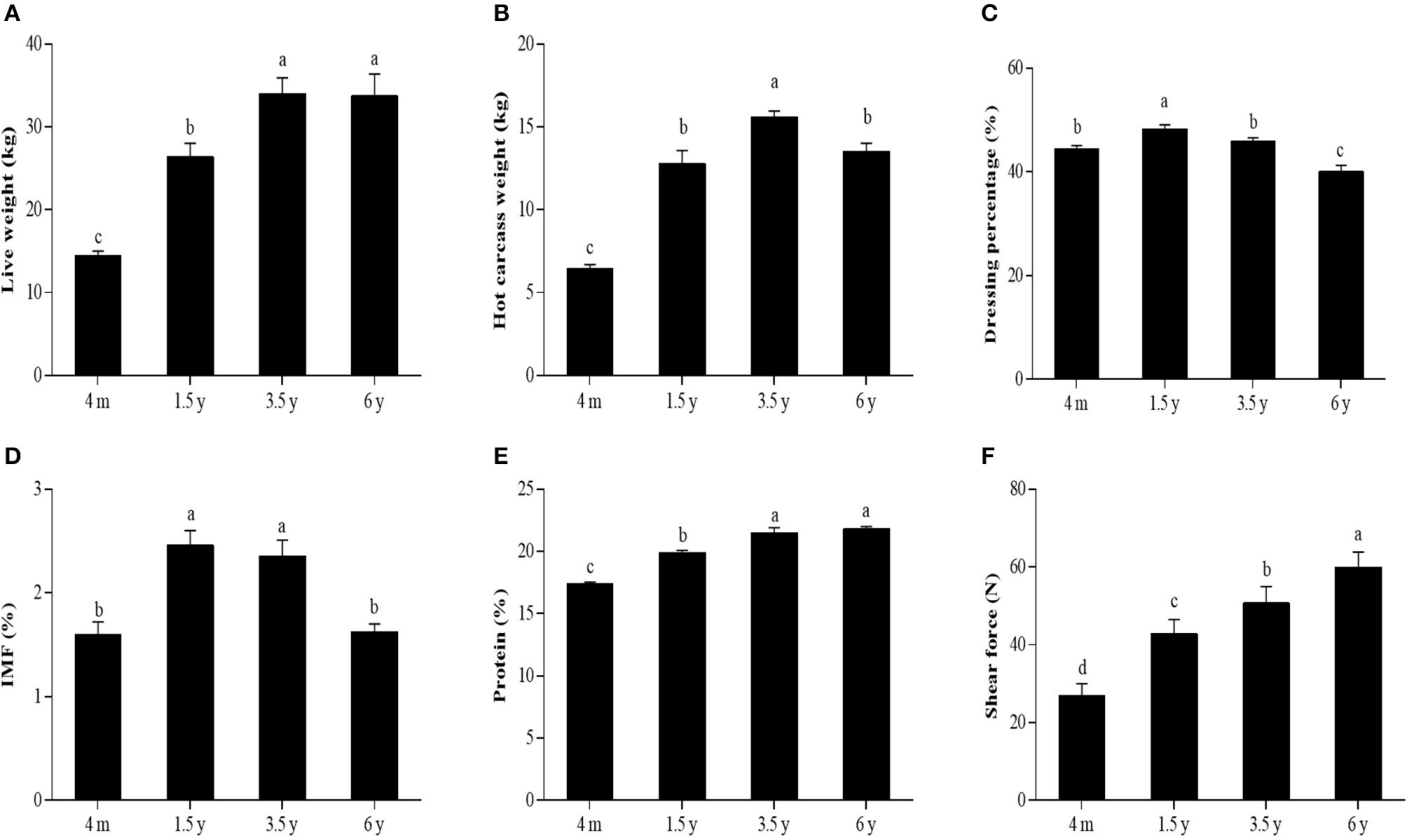
The slaughter performance and meat quality of Tibetan sheep at different stages. **(A)** Live weight. **(B)** Hot carcass weight. **(C)** Dressing percentage. **(D)** IMF content. **(E)** Protein content. **(F)** The shear force of LT muscle of Tibetan sheep at 4 m, 1.5 y, 3.5 y, and 6 y. Data were represented as the means ± SD. Different letters (a–d) indicated significant difference between the different age groups (*p* < 0.05).

The slaughter performance and meat quality of the Tibetan sheep at different stages are shown in Figure 1. Live weight and protein content was found to significantly increase from 4 m to 3.5 y (*p* < 0.05) but became insignificant from 3.5 y to 6 y (Figures 1A,E). Hot carcass weight was significantly increased from 4 m to 3.5 y (*p* < 0.05), while the weight was significantly decreased from 3.5 to 6 y (*p* < 0.05) (Figure 1B). Dressing percentage was significantly higher in the 1.5 y than other groups (*p* < 0.05) (Figure 1C). IMF content was significantly higher in the 1.5 y and 3.5 y than 4 m and 6 y (*p* < 0.05). However, there was no significant difference in the IMF content between 1.5 y and 3.5 y (Figure 1D). The shear force was significantly increased with the increase of age (*p* < 0.05) (Figure 1F).

### Overview of circRNA Sequencing

**Figure 2:**
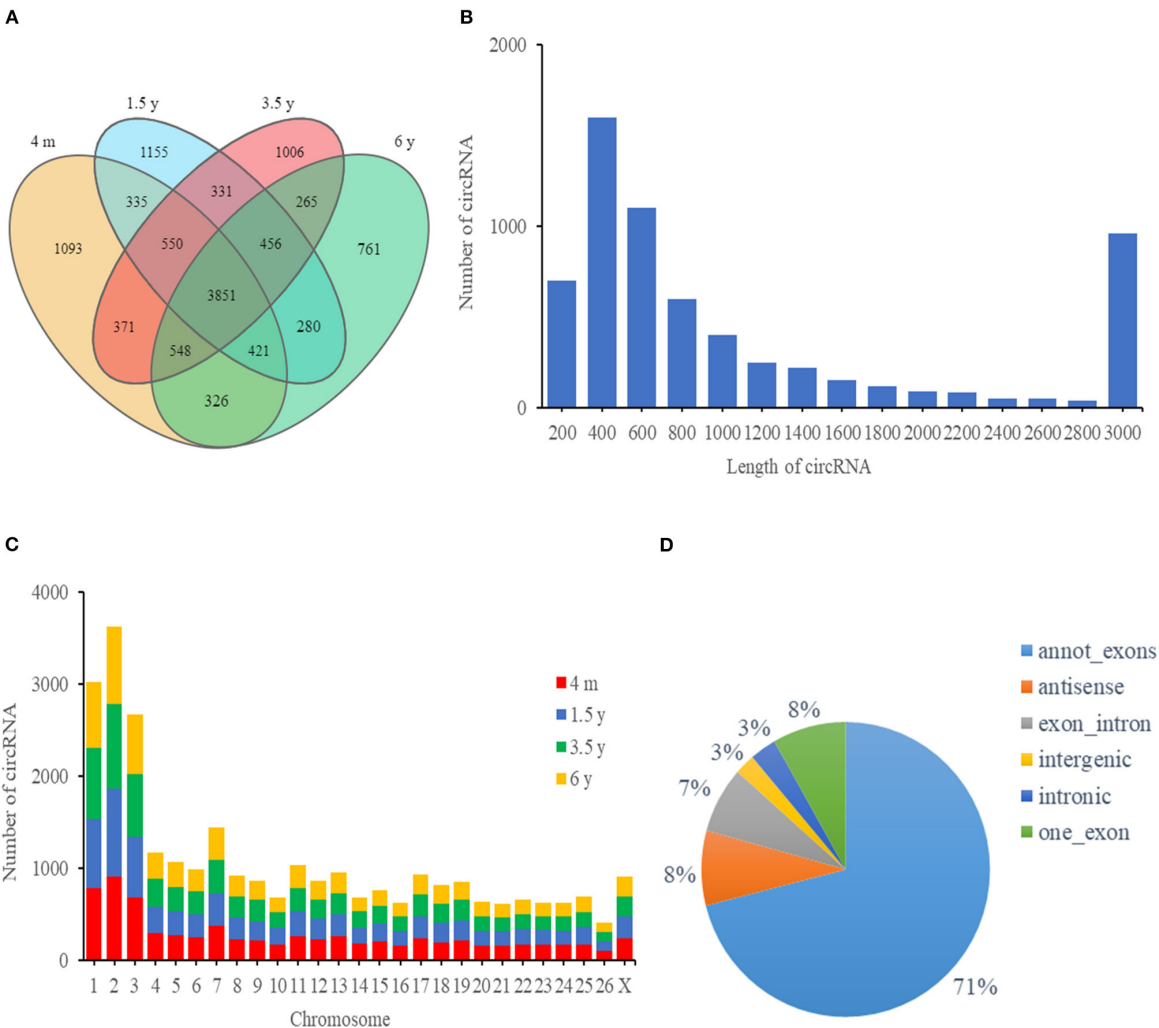
circRNA characteristics of the LT muscle of Tibetan sheep. **(A)** Venn diagram showing the overlap of annotated circRNAs of Tibetan sheep among 4 m, 1.5 y, 3.5 y, and 6 y. **(B)** The length distribution of circRNAs. **(C)** Chromosome distribution of circRNA in the LT muscle of Tibetan sheep. **(D)** The distribution of genomic regions from which circRNAs were derived.

A total of 1,451,204,326 high-quality clean reads were obtained after filtering the raw data. A total of clean reads (90,738,055 from 4 m group, 94,864,497 from 1.5 y group, 89,970,023 from 3.5 y group, and 87,228,505 from 6 y group) were obtained. About 2.43, 5.50, 7.95, and 11.27% clean reads did not match the sheep reference genome in 4 m, 1.5 y, 3.5 y, and 6 y of Tibetan sheep, respectively. A total of 11,749 circRNAs comprising 3,851 circRNAs co-expressed in the four groups (4 m, 1.5 y, 3.5 y, and 6 y) (Figure 2A). The naming and numbering of these circRNAs range from circ_000001 to circ_011749. According to the location in the sheep genome, most circRNAs were derived from gene encoding exons, and the rest were derived from antisense strands and introns (Figure 2D). Most circRNAs were between 200 and 800 bp in length, and the number of circRNAs with a length of 400 bp was the largest (Figure 2B). These circRNAs were distributed in 26 autosomes and X chromosomes, mainly in chromosomes 1, 2, and 3 (Figure 2C).

### Differential Expression Analysis

**Figure 3:**
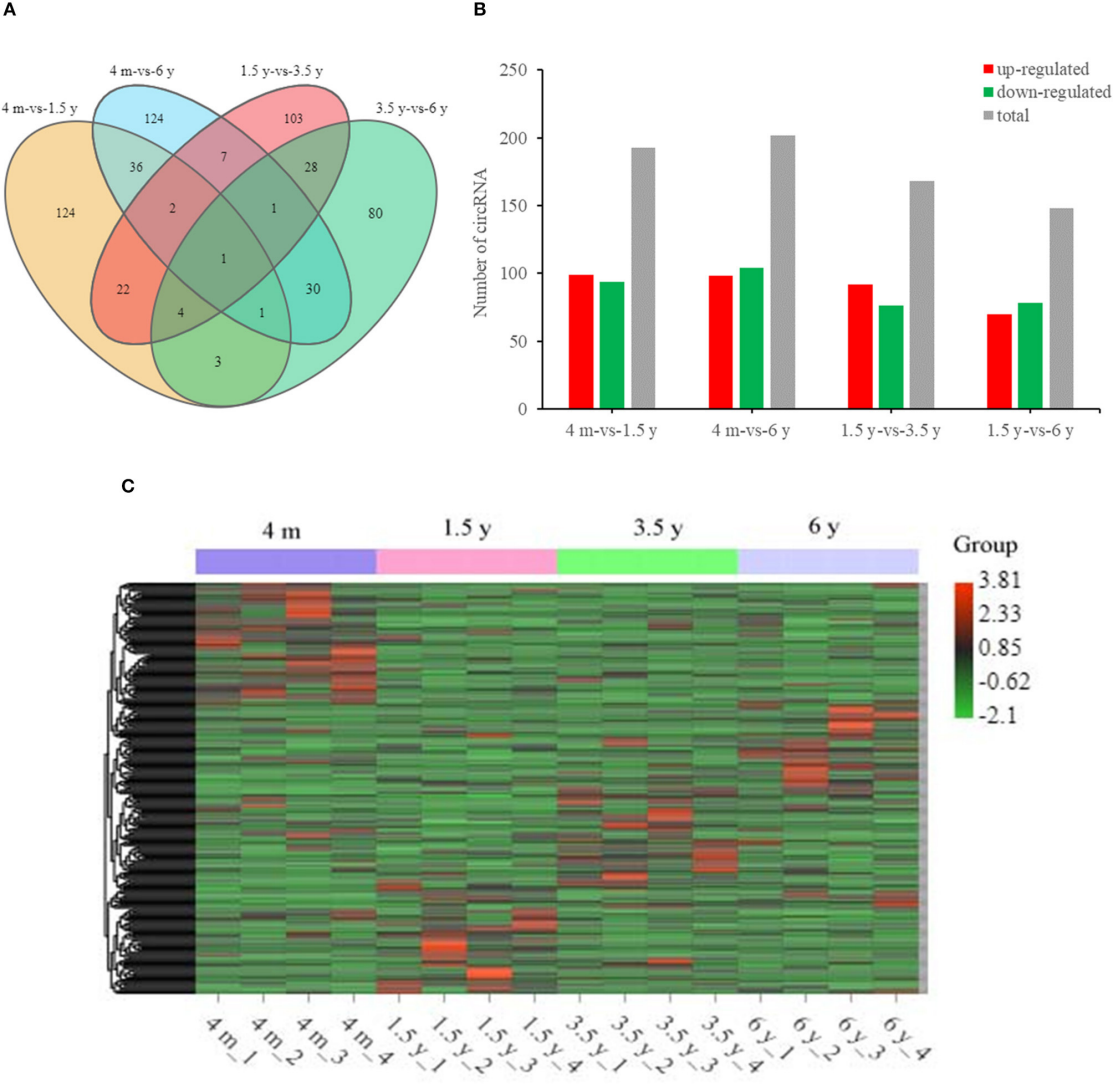
Summary of circRNA differential expression analysis. **(A)** The Venn diagrams of the shared and unique differential circRNAs in the four comparative groups. **(B)** Statistics of DE circRNA in the four comparative groups. **(C)** Cluster heat map of DE circRNA expression. Red means upregulation, green means downregulation.

Overall, there were 193 (99 upregulated; 94 downregulated), 168 (92 upregulated; 76 downregulated), 148 (70 upregulated; 78 downregulated), and 202 (98 upregulated; 104 downregulated) DE circRNAs that were identified in the 4 m vs. 1.5 y, 1.5 y vs. 3.5 y, 3.5 y vs. 6 y, and 4 m vs. 6 y groups, respectively (Figures 3A–C). There was a circRNA co-expressed in the four contiguous period transcriptome comparative groups (Figure 3A).

### GO Enrichment and KEGG Pathway Analysis for Source Genes of DE circRNAs

**Figure 4:**
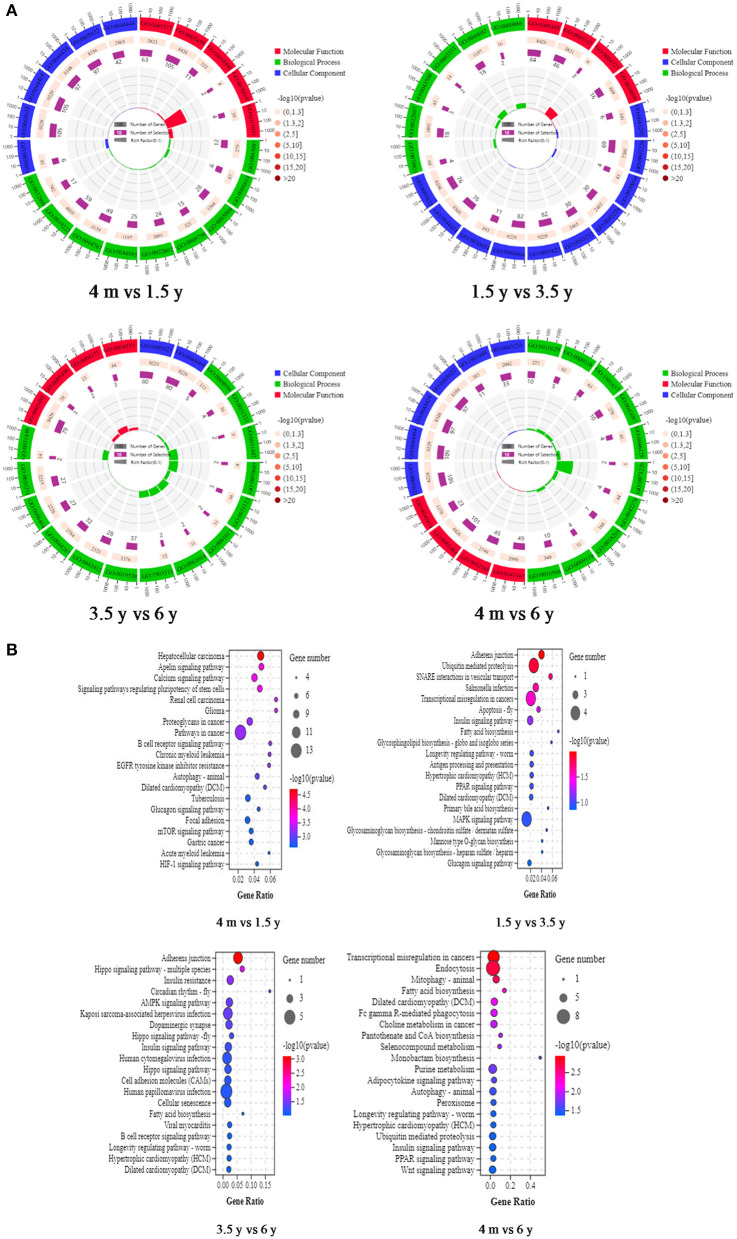
GO functional annotation and KEGG enrichment analysis of source genes of DE circRNAs. **(A)** Representative of the GO enrichment circle diagram in four contiguous period transcriptome comparative groups (4 m vs. 1.5 y, 1.5 y vs. 3.5 y, 3.5 y vs. 6 y, and 4 m vs. 6 y group). Green is biological process (BP). Red is molecular function (MF). Blue is cellular component (CC). **(B)** Representative KEGG pathway analysis of the source genes of DE circRNAs in 4 m vs. 1.5 y, 1.5 y vs. 3.5 y, 3.5 y vs. 6 y, and 4 m vs. 6 y group, respectively.

In this study, 192 of the 193 DE circRNAs in the 4 m vs. 1.5 y group were identified to be derived from 176 source genes, and the remaining 1 circRNA was derived from the intergenic region without the source genes. In the 1.5 y vs. 3.5 y group, 160 of the 168 DE circRNAs identified were found to be derived from 154 source genes, and the remaining 8 circRNA were from intergenic regions without the source genes. In the 3.5 y vs. 6 y group, 145 of the 148 DE circRNAs identified were derived from 141 source genes, and the remaining 3 circRNA were from intergenic regions without source genes. In the 4 m vs. 6 y group, 195 of 202 DE circRNAs identified were derived from 181 source genes, and the remaining 7 circRNA were from the intergenic regions without the source genes. To further analyze the biological function of the circRNAs, GO enrichment and KEGG pathway analysis were performed on the source genes of circRNA. A total of 323, 172, 160, and 213 GO terms were enriched of 4 m vs. 1.5 y, 1.5 y vs. 3.5 y, 3.5 y vs. 6 y, and 4 m vs. 6 y group, respectively. In the 4 m vs. 1.5 y group, the most source genes of DE circRNAs were significantly enriched into protein binding (GO:0005515), binding (GO:0005488), myofibril (GO:0030016), and regulation of GTPase activity (GO:0043087). At this stage, the protein and energy metabolism were more active in the Tibetan sheep, which might further regulate muscle growth and energy metabolism. In the 1.5 y vs. 3.5 y group, binding (GO:0005488), contractile fiber (GO:0043292), and organelle assembly (GO:0070925) comprised the significantly enriched GO terms. At this stage, the organ assembly and muscle fiber contraction might be related to the transformation of muscle fiber types. In the 3.5 y vs. 6 y group, the most source genes of the DE circRNAs were significantly enriched into the binding (GO:0005488), cell (GO:0005623), and positive regulation of the catabolic process (GO:0009896). At this stage, this process was related to the regulation of catabolism, and the IMF content decreased during this process. Negative regulation of the gene expression (GO:0010629) and ion binding (GO:0043167) cell (GO:0005623) comprised the main GO terms, which were significantly enriched in the source genes of DE circRNAs in the 4 m vs. 6 y group (Figure 4A). The KEGG analysis results indicated that source genes of DE circRNAs were significantly enriched in the Apelin signaling pathway, calcium signaling pathway, and signaling pathways regulating pluripotency of stem cells in the 4 m vs. 1.5 y group. The 1.5 y vs. 3.5 y group was significantly enriched in adherens junction, ubiquitin mediated proteolysis, and SNARE interactions in vesicular transport. The 3.5 y vs. 6 y group was significantly enriched in adherens junction, Hippo signaling pathway-multiple species, and AMPK signaling pathway, and these signaling pathways play an important role in muscle growth and development. The 3.5 y vs. 6 y group showed significant enrichment in endocytosis, mitophagy-animal, and fatty acid biosynthesis (Figure 4B).

### Construction of circRNA–miRNA–mRNA Regulatory Network

**Figure 5:**
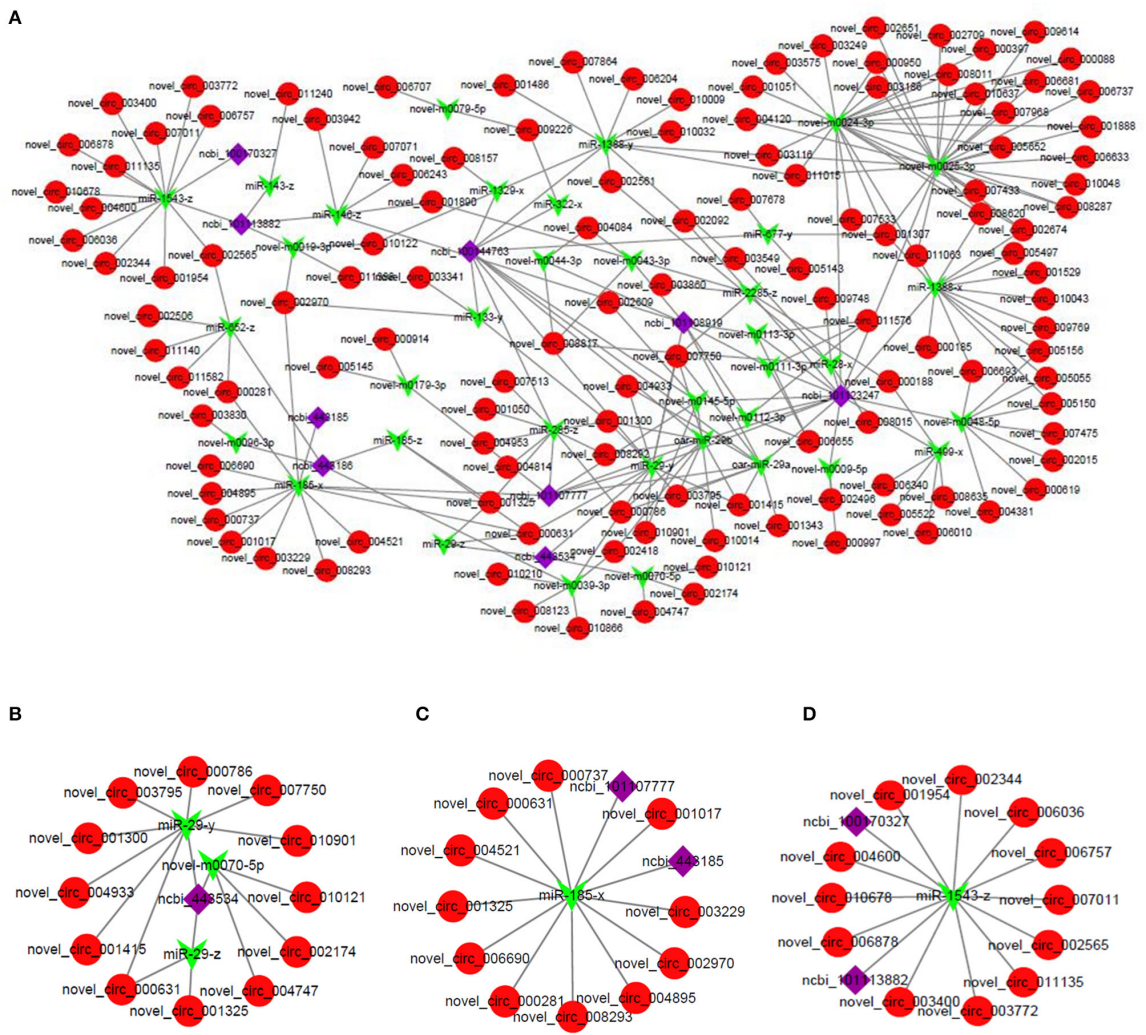
**(A)** The circRNA–miRNA–mRNA interaction network in LT muscle of Tibetan sheep. **(B)** Representative subnetwork of *LEP*. **(C)** Representative subnetwork of *SCD*. **(D)** Representative subnetwork of *FASN*. Red circles represent circRNAs, green V shape represents miRNAs, and purple squares represent genes.

To further analyze the biological functions of these DE circRNAs, based on the ceRNA hypothesis mechanism, a regulatory network of circRNA–miRNA–mRNA interaction was constructed by integrating the results of our previous miRNA and mRNA sequencing data. The ceRNA regulatory network contained 197 circRNA–miRNA pairs and 53 miRNA–mRNA pairs and included 128 circRNAs, 36 miRNAs, and 9 mRNAs (Figure 5A). The results of our previous mRNA sequencing revealed that among them, the ncbi_443534 (*LEP*), ncbi_443185 (*SCD*), and ncbi_100170327 (*FASN*) were all important genes in the AMPK signaling pathway. AMPK signaling pathway was evident in the 4 m vs. 1.5 y group, 1.5 y vs. 3.5 y group, 3.5 y vs. 6 y group, and 4 m vs. 6 y group, respectively (Supplementary Figures 1A–D). Therefore, these genes might be related to the transformation of the muscle fiber types and further influence the meat quality of Tibetan sheep. The subnetworks of ncbi_443534 (*LEP*) is shown in Figure 5B; it showed that the expression of *LEP* was regulated by 12 circRNAs and 3 miRNAs. Among them, the most significant difference was novel_circ_004933 and miR-29-y. The subnetworks of ncbi_443185 (*SCD*) is shown in Figure 5C, where the expression of *SCD* was found to be regulated by 11 circRNA and 1 miRNA. The subnetworks of ncbi_100170327 (*FASN*) is shown in Figure 5D. The expression of FASN was regulated by 12 circRNA and 1 miRNA.

### Validation of circRNA Expression by qPCR

**Figure 6:**
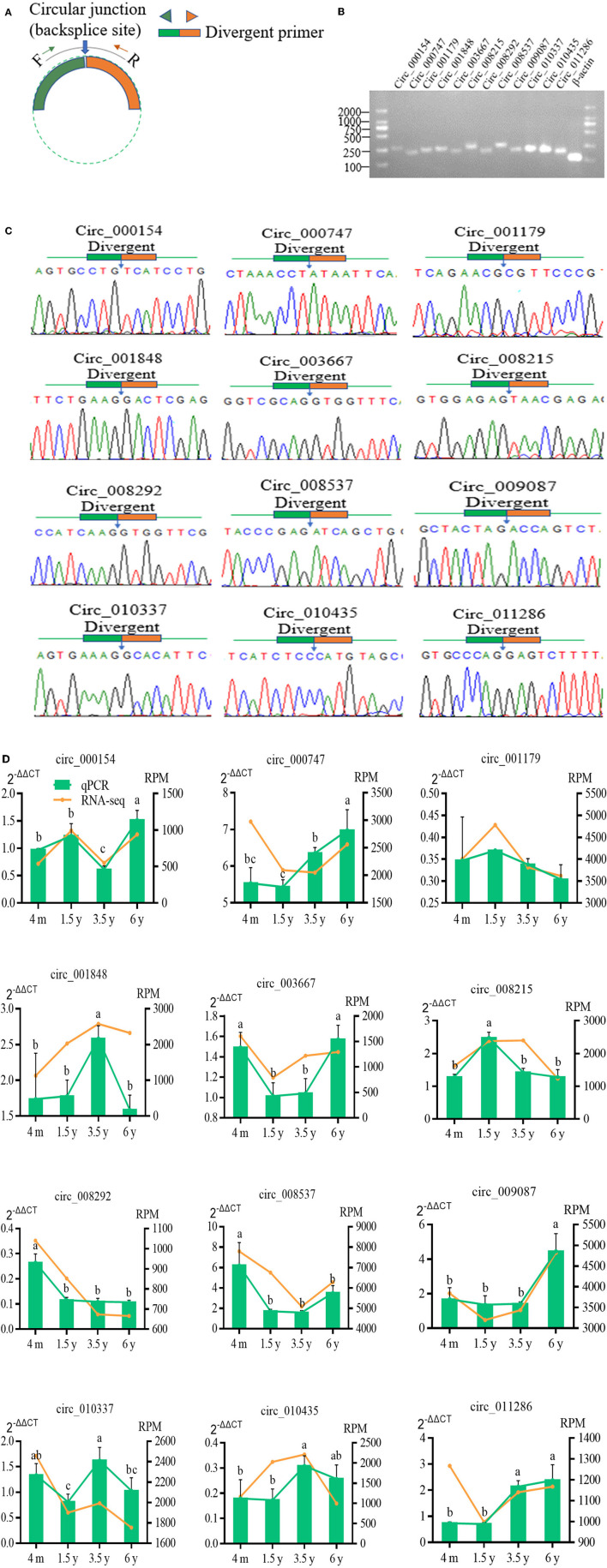
Verification of 12 randomly selected circRNAs from RNA-Seq. **(A)** Divergent primers were designed used to circRNA verification. **(B)** PCR amplification of circRNAs with divergent primers. PCR products were visualization analyzed on a 1.5% gel electrophoresis. **(C)** Circular junctions were confirmed by Sanger sequencing of RT-PCR products using divergent primers. **(D)** Comparison of the expression levels of circRNA between RNA-Seq and RT-qPCR. RT-qPCR data were shown as the means ± SD. Different letters (a–c) indicated significant difference between different ages (*p*<0.05).

Twelve DE circRNAs were randomly selected and the divergent primers containing their junction sites were synthesized to determine the presence of these circRNAs, and to verify the authenticity of the predicted circRNAs and the reliability of the RNA-Seq data (Figure 6A). The RT-qPCR results indicated the expression profiles of these 12 circRNAs to be consistent with the RNA sequencing results (Figure 6D). A 1.5% agarose gel electrophoresis indicated the size of a single band of each selected circRNA to be consistent with the expected size (Figure 6B). The qPCR products fragment was then sequenced using Sanger sequencing, and the results indicated the circular junction sequence sites of these circRNA obtained by Sanger sequencing to be completely consistent with the results of the circRNA sequencing (Figure 6C). This showed the reliability of the sequencing data and expression of the circRNA identified in this study.

#### The Relationship Between the circRNAs and Slaughter Performance and Meat Quality

**Figure 7:**
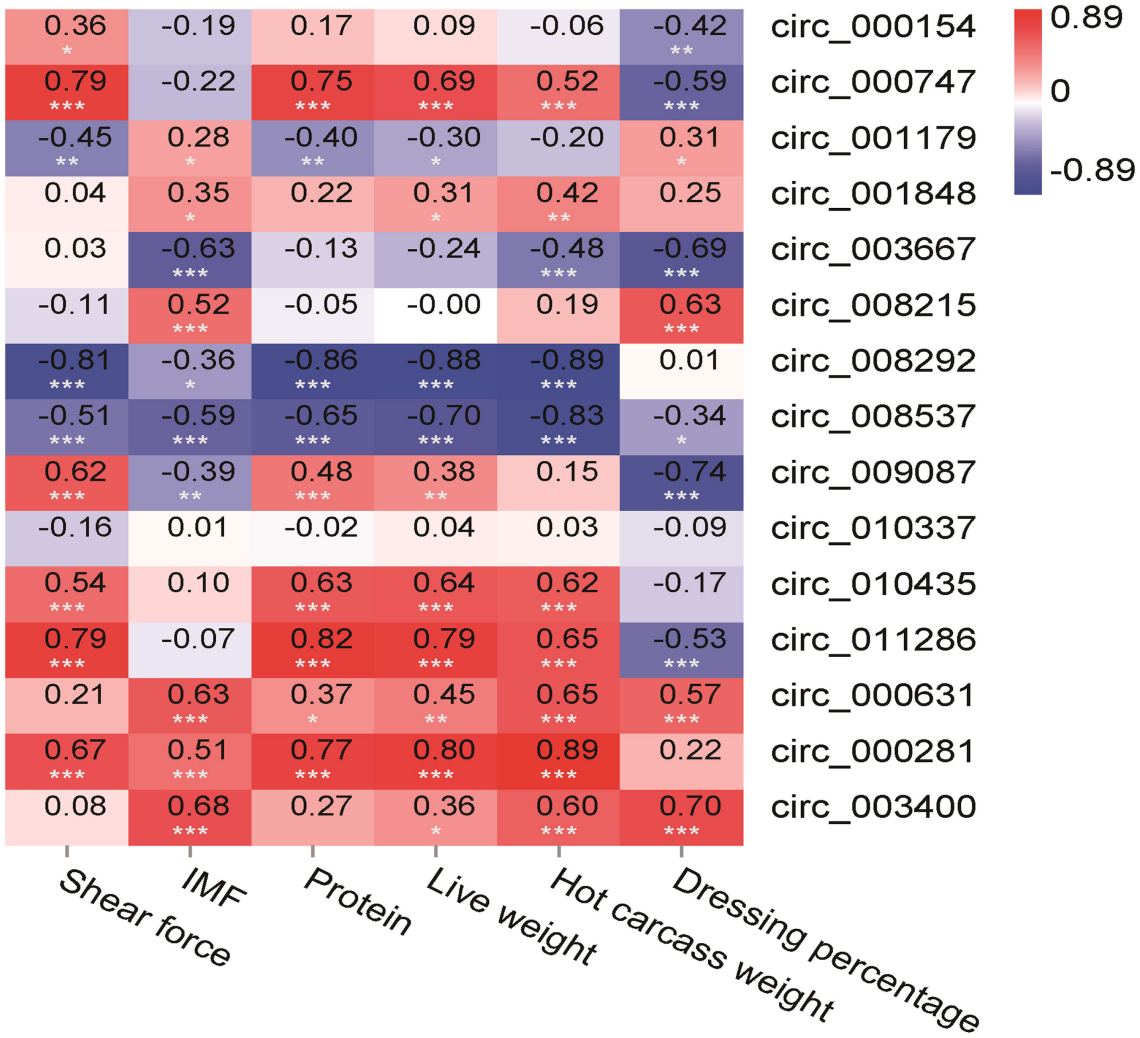
Pearson correlations between expression of circRNAs and slaughter performance and meat quality in the LT muscle of Tibetan sheep. *Correlation is significant at *p* < 0.05, **correlation is significant at *p* < 0.01, ***correlation is significant at *p* < 0.001.

In this study, the slaughter performance and meat quality were selected and the correlation analysis was performed with the 12 circRNAs to analyze the functions of circRNAs in the LT muscle growth and development. A significant correlation was found between the most slaughter performance and meat quality and circRNAs (Figure 7). There was a positive correlation between the shear force and circ_000747 (0.79, *p* < 0.001), circ_009087 (0.62, *p* < 0.001), circ_010435 (0.54, *p* < 0.001), and circ_011286 (0.79, *p* < 0.001). On the contrary, there was a negative correlation between the shear force and circ_008292 (−0.81, *p* < 0.001). A correlation was found between IMF and circ_003667 (−0.63, *p* < 0.001), and between IMF and circ_008215 (0.52, *p* < 0.001). There was a positive correlation between the protein and circ_000747 (0.75, *p* < 0.001), circ_010435 (0.63, *p* < 0.001), and circ_011286 (0.82, *p* < 0.001). A negative correlation was found between the protein and circ_008292 (−0.86, *p* < 0.001). On the other hand, there was a positive correlation between the live weight and circ_000747 (0.69, *p* < 0.001), circ_010435 (0.64, *p* < 0.001), and circ_011286 (0.79, *p* < 0.001). There was a negative correlation between the live weight and circ_008292 (−0.88, *p* < 0.001) and circ_008537 (−0.70, *p* < 0.001). A positive correlation was found between the hot carcass weight and circ_010435 (0.62, *p* < 0.001) and circ_011286 (0.65, *p* < 0.001), while there was a negative correlation between the hot carcass weight and circ_008292 (−0.89, *p* < 0.001). There was a positive correlation between the dressing percentage and circ_008215 (0.63, *p* < 0.001). A negative correlation was found between the dressing percentage and circ_009087 (−0.74, *p* < 0.001).

## Discussion

Studying the mechanism of skeletal muscle growth and development can be useful for improving the meat yield and meat quality. The skeletal muscle accounts for 40–50% of an animal's body weight (7). The skeletal muscle is formed in the embryo paraxial mesoderm in mammals before birth, and the myoblasts differentiate into myotubes to form myofiber (34, 35). The skeletal muscle growth and development after birth are mainly based on the increase in the muscle fiber diameter and the transformation of the muscle fiber types (36, 37). Our previous study has found the 1.5 y Tibetan sheep meat was more suitable for a healthy human diet (38) with more meat tenderness, higher IMF content, abundant unsaturated fatty acids, and more content of type I muscle fiber. Therefore, 1.5 y is the best age for slaughter of Tibetan sheep. *LEP, SCD, LIPE, ADIPOQ*, and *FASN* have been found to participate in the AMPK signaling pathway and regulate the muscle growth and development.

CircRNAs are abundant non-coding RNAs in the transcriptome of eukaryotes, which are important regulatory factors of transcription and post-transcriptional regulation of gene expression. The RNase R treatment was used to remove the linear RNAs for obtaining circRNAs. Jeck et al. (39) have pointed out that after the RNase R treatment, the circRNA remains intact but efficiently degrades most linear RNAs, and without the RNase R treatment gives a set of tenfold fewer circRNAs. At the same time, the circRNAs also regulate the function of miRNA at the translation level (40). Legnini et al. (41) and Wei et al. (14) found that the expression of the genes associated with the skeletal muscle growth and development. Du et al. (42) also found the circRNA combined with the RNA-binding proteins to play an important role in animal muscle development. At the same time, the circRNAs can combine with the miRNA to weaken the function of miRNA and further regulate gene expression (43). In this study, 72.85% clear reads of the LT muscle of Tibetan sheep at four age groups were found to match with the sheep reference genome, which was higher than 57.31% clear reads in the study of sheep skeletal muscle by Cao et al. (44). The distribution of the circRNA types showed 71% to be derived from multiple exons, while Cao et al. (44) have found 88.94% of circRNAs to be derived from multiple exons in the sheep skeletal muscle. In this study, the distribution of circRNA was related to the number of parental genes on the sheep chromosomes. For instance, there were maximal genes on chromosome 2 and the least on chromosome 26 in the sheep (45). A total of 11,749 circRNAs were identified in this study, which was higher than the 886 circRNAs in the study of Cao et al. (44). In this study, a total of 711 DE circRNAs have been identified in the four contiguous period transcriptome comparative groups with a |log2FC|>1 and *p* < 0.05. Huang et al. (46) have identified 1,128 DE circRNAs in the skeletal muscle of the two breeds of cattle. The circRNA sequencing data were verified by qPCR analysis and Sanger sequencing, showing that the circRNA obtained in this study was reliable.

To analyze the role of the circRNAs in the skeletal muscle growth and development of Tibetan sheep, GO function annotation and KEGG enrichment analysis were performed on the source genes of DE circRNAs. GO function annotations results showed these source genes of circRNA to be mainly involved in biological processes such as developmental processes, organ growth, muscle adaptation, protein metabolism processes, and negative regulation of gene expression. These source genes of circRNAs participate in the cell cycle progression and cell development and thus participate in regulating muscle growth and development. For example, *MAPK1* can mediate a series of biological reactions in the cells, including cell proliferation, differentiation and apoptosis, and so on (47). The *IGF1* insulin signaling inhibits proteolysis and promotes muscle growth (48). ACTA1 is the skeletal muscle actin A1, the main component of the muscle fibers, with an important role in muscle contraction (49). Muscle growth and development is a complex biological process, and the function of the source genes of these circRNAs indicated the circRNAs also play an important role in the mammalian skeletal muscle growth and development after birth.

Moreover, the KEGG analysis was conducted on the source genes of DE circRNAs to further analyze the functions of these circRNAs. In the 4 m vs. 1.5 y group, there was mainly significant enrichment in the Apelin signaling pathway and calcium signaling pathway. The 1.5 y vs. 3.5 y group was mainly significantly enriched in the insulin signaling pathway and fatty acid biosynthesis. In the 3.5 y vs. 6 y group, there was mainly significant enrichment in the Hippo signaling pathway and AMPK signaling pathway. The 4 m vs. 6 y group showed main significant enrichment in the PPAR signaling pathway and fatty acid biosynthesis. These signaling pathways are all related to muscle growth and development (50, 51). *MAPK1* mainly participates in the Apelin signaling pathway regulating cell proliferation, differentiation, and apoptosis. *PHKA1* might be involved in the insulin signaling pathway for regulating the absorption of glucose in the skeletal muscle (52), to further regulate the energy metabolism of the skeletal muscles. *PFKFB4* was enriched in the AMPK signaling pathway, which regulates the level of glycolysis in the cell (53). The level and type of glucose metabolism in the cells are related to the muscle fiber types. PFKFB4 might be involved in regulating the transformation of skeletal muscle fiber types, which further influence the meat quality. *ACACB* in the fatty acid biosynthesis signaling pathway has been related to the synthesis of the fatty acids; it is a rate-limiting enzyme promoting fat synthesis and playing an important role in glycolipid metabolism (54). The analyses of the KEGG enrichment indicated that the source genes of circRNA are mainly enriched in the signaling pathways related to muscle development.

Many previous studies have shown one of the main functions of circRNA to act as a miRNA sponge for indirectly regulating the expression of the downstream target genes of miRNA (55). Hansen et al. (56) found ciRS-7 to bind to miR-7, reducing the miR-7 activity, and increasing the expression level of the miR-7 target genes. Herein, this study analyzed the circRNA–miRNA–mRNA ceRNA interaction network combining the previous miRNA-seq and mRNA-seq data, and further revealing the function of these DE circRNAs in the skeletal muscle growth and development of Tibetan sheep. The results indicated 197 circRNA–miRNA pairs and 53 miRNA–mRNA pairs, including 128 circRNAs, 36 miRNAs, and 9 mRNAs in the ceRNA interaction network. Among them, *LEP, SCD*, and *FASN* were all related to muscle growth and development (57–59). Previous studies have found that *LEP, SCD*, and *FASN* participate in the AMPK signaling pathway, and similar results were found in our study of LT muscle of Tibetan sheep at different growth stages, which might be related to the energy metabolism and transformation of the muscle fiber types in Tibetan sheep. Also, some non-coding RNAs, like miR-29-3p, miR-29-z, and novel-m0070-5, were identified in the ceRNA regulatory network. *LEP* was predicted to be regulated by the circ_000786/circ_007750/circ_003795-miR-29-3p signals. *SCD* was predicted to be regulated by circ_006690/circ_004521-miR-185-5p signals. *FASN* was predicted to be regulated by circ_002344/circ_006036-miR-1543-z signals. miR-29-3p includes miR-29a-3p, miR-29b-3p, and miR-29c-3p (60, 61). Wu et al. (62) demonstrated that the lncRNA promotes the proliferation of the skeletal muscle satellite cells in the Hu sheep by regulating the miR-29a. Li et al. (63) also found miR-29 to inhibit the proliferation of the rhabdomyosarcoma cells by inhibiting the expression of the cell cycle–related genes. MiR-29c can improve the quality and function of skeletal muscles by inhibiting atrophy-related genes through the proliferation and differentiation of the muscle cells (64). Sun et al. (65) found circRNA cirbr4 to induce the proliferation and migration of the vascular smooth muscle cells by binding miR-185-5p. However, there is no study on the miR-1543 now. Therefore, the circRNA obtained in this study might serve as a miRNA sponge, which has a critical role in transforming of the form of energy metabolism in the skeletal muscles of the Tibetan sheep to adapt to the harsh environment of the Qinghai–Tibet Plateau. The energy metabolism in the skeletal muscle was dominated by glycolysis with the increase of age, and the oxidized muscle fibers were gradually transformed into glycolytic muscle fibers. There was an increase in the content of the glycolytic muscle fibers, while the number of mitochondria and hemoglobin in glycolytic muscle fibers was reduced, the content of IMF was decreased, and the diameter of the muscle fibers was larger, which would consequently lead to poor tenderness of Tibetan sheep meat. Correlation analysis indicated significant correlation between the circRNAs and meat quality and slaughter performance.

In conclusion, the dynamic expression profile and differential expression information of the circRNAs were obtained through RNA sequencing and bioinformatics analysis, The DE circRNA was found to possibly have an important role in the development of skeletal muscle in Tibetan sheep after birth by regulating the expression level of the source genes and as ceRNAs. The results of the present study of the ceRNA regulatory network indicated that circ_000631, circ_000281, and circ_003400 regulate the expression of the *LEP, SCD*, and *FASN* genes in the AMPK signaling pathway by combining with miR-29-3p, miR-185-5p, and miR-1543-z. This would change the form of energy metabolism in the skeletal muscle, gradually transforming the oxidized muscle fibers into glycolytic muscle fibers. decreased the IMF content, but the diameter of muscle fibers increased, reducing the tenderness of Tibetan sheep meat. In the production of Tibetan sheep, if these sheep were slaughtered in time, their meat quality is the best due to the characteristics of skeletal muscle growth and development at 1.5 y, which can be used to meet the consumers' demand for high-quality mutton. This study provides a new idea for in-depth exploration of the muscle development mechanism of the Tibetan sheep.

## Data Availability Statement

The datasets presented in this study can be found in online repositories. The names of the repository/repositories and accession number(s) can be found at: NCBI [accession: PRJNA780979] and [accession: PRJNA781754].

## Ethics Statement

The animal study was reviewed and approved by the Faculty Animal Policy and Welfare Committee of Gansu Agricultural University (Ethic approval file No. GSAU-Eth-AST-2021-001).

## Author Contributions

GB and YL conceived and designed the study. GB, FZ, XL, BS, JW, JH, and YW collected the samples. GB, LZ, JW, and YW performed the experiments and analyzed the data. GB wrote the manuscript. YL, SL, and JW contributed to the revisions of the manuscript. All authors read and approved the manuscript.

## Funding

This research was funded by the fund of Distinguished Young Scholars Fund of Gansu Province (21JR7RA857), the Fuxi Young Talents Fund of Gansu Agricultural University (Gaufx-03Y04), and the Projects of Gansu Agricultural University (GSAU-ZL-2015-033), Key R&D Projects in Gansu Province (18YF1WA082).

## Conflict of Interest

The authors declare that the research was conducted in the absence of any commercial or financial relationships that could be construed as a potential conflict of interest.

## Publisher's Note

All claims expressed in this article are solely those of the authors and do not necessarily represent those of their affiliated organizations, or those of the publisher, the editors and the reviewers. Any product that may be evaluated in this article, or claim that may be made by its manufacturer, is not guaranteed or endorsed by the publisher.
