# Supplementary material for: Characterization of the circRNA–miRNA–mRNA Network to Reveal the Potential Functional ceRNAs Associated With Dynamic Changes in the Meat Quality of the Longissimus Thoracis Muscle in Tibetan Sheep at Different Growth Stages
Source: Front Vet Sci. 2022 Apr 1;9:803758. doi: 10.3389/fvets.2022.803758 (PMC9011000; doi:10.3389/fvets.2022.803758)
Supplement: Supplementary file 1 [file Data_Sheet_1.docx]

Fig. S1. AMPK signaling pathway. The white square represents a gene or protein, the red square is the up-regulated genes in the pathway, the green square is the down-regulated genes in the pathway. (A-D) AMPK signaling pathway in the 4 m vs 1.5 y group 1.5 y vs 3.5 y group, 3.5 y vs 6 y group, 4 m vs 6 y group, respectively.
