# Supplementary figures and images for: Characterization of the circRNA–miRNA–mRNA Network to Reveal the Potential Functional ceRNAs Associated With Dynamic Changes in the Meat Quality of the Longissimus Thoracis Muscle in Tibetan Sheep at Different Growth Stages
Source: Front Vet Sci. 2022 Apr 1;9:803758. doi: 10.3389/fvets.2022.803758 (PMC9011000; doi:10.3389/fvets.2022.803758)

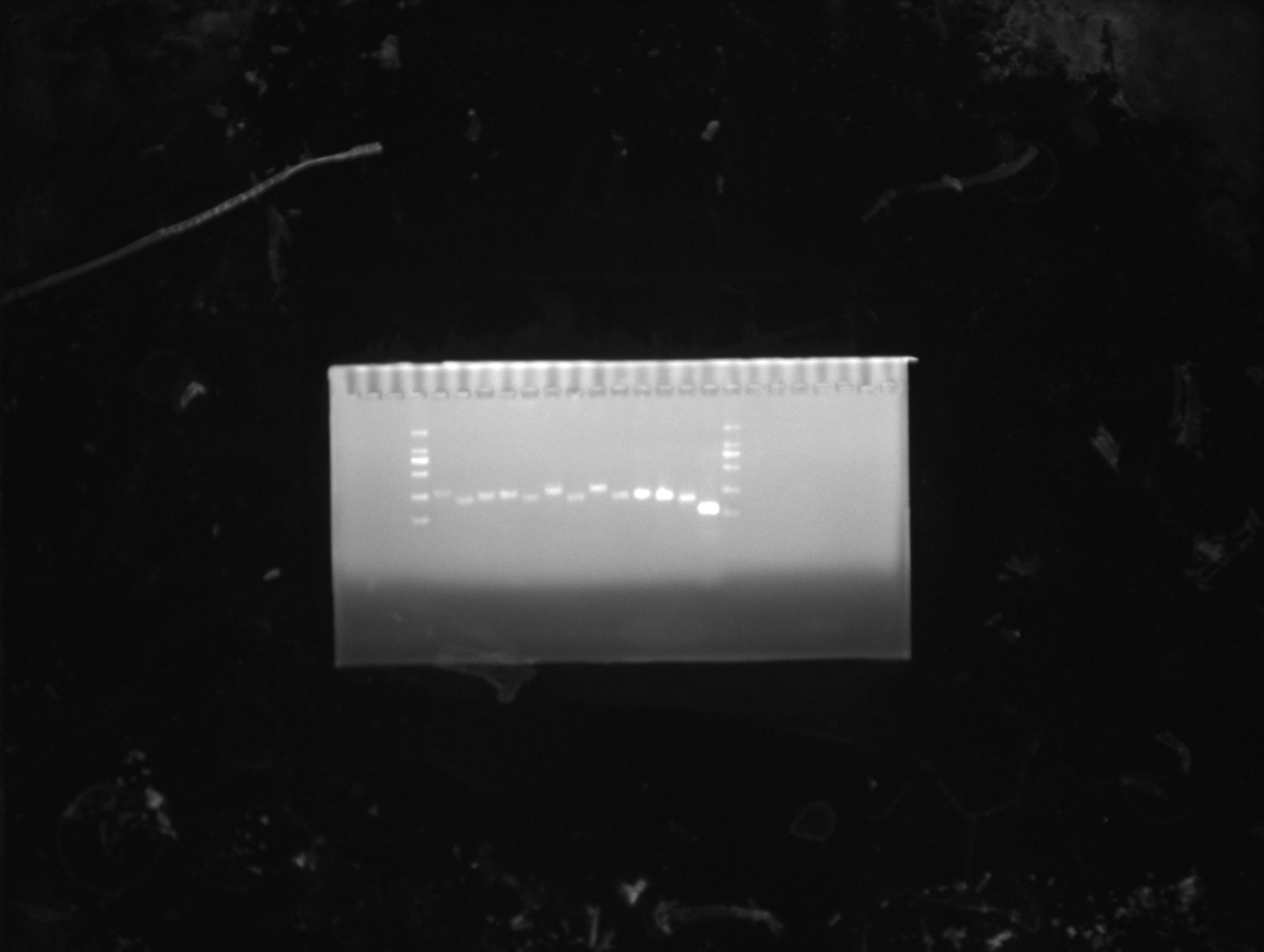

Supplement: Supplementary file 2 [file Image_1.TIF]
